# Dispositional mindfulness, emotional regulation and perceived stress among nursing students

**DOI:** 10.1590/1980-220X-REEUSP-2022-0086en

**Published:** 2022-08-05

**Authors:** Daiana Alves Vendramel da Costa, Moisés Kogien, Shaiana Vilella Hartwig, Gímerson Erick Ferreira, Michelly Kim de Oliveira Rosa Guimarães, Mara Regina Rosa Ribeiro

**Affiliations:** 1 Universidade Estadual de Mato Grosso, Faculdade de Ciências da Saúde, Cáceres, MT, Brazil.; 2 Universidade Federal de Mato Grosso, Faculdade de Enfermagem, Cuiabá, MT, Brazil.

**Keywords:** Education, Nursing, Mindfulness, Emotional Regulation, Stress, Psychological, Cognitive Neuroscience, Educación en Enfermería, Atención Plena, Regulación Emocional, Estrés Psicológico, Neurociencia Cognitiva, Educação em Enfermagem, Atenção Plena, Regulação Emocional, Estresse Acadêmico, Neurociência Cognitiva

## Abstract

**Objective:**

To investigate the correlation between dispositional mindfulness, emotional regulation and perceived stress and to verify factors associated with dispositional mindfulness among nursing students.

**Method:**

A correlational, cross-sectional study with public undergraduate students. The following instruments were used: Mindful Attention Awareness Scale, Emotional Regulation Questionnaire and Perceived Stress Scale. Analyzes were performed using t tests, Pearson’s correlation and multiple linear regression.

**Results:**

The study included 330 students. There was no correlation between dispositional mindfulness and general emotional regulation score and/or perceived stress and a weak correlation with the emotional regulation dimension emotional suppression. Being in psychological treatment and psychoactive substance use were associated with decreased dispositional mindfulness. Age, sufficient sleep hours and emotional suppression were associated with an increase in this variable.

**Conclusion:**

There was a relationship between dispositional mindfulness only with emotional suppression, in addition to the connection of this variable with the perception of sufficient sleep hours, age, use of alcohol or psychoactive substances, undergoing psychological/psychiatric follow-up and emotional suppression.

## RESUMO

**Objetivo:**

Investigar a correlação entre atenção plena disposicional, regulação emocional e estresse percebido e verificar fatores associados à atenção plena disposicional em estudantes de enfermagem.

**Método:**

Estudo correlacional, transversal, com estudantes de universidade pública. Foram utilizados os instrumentos: *Mindful Attention Awareness Scale*, *Emotional Regulation Questionnaire* e *Perceived Stress Scale*. Análises foram realizadas por meio de testes t, correlação de Pearson e regressão linear múltipla.

**Resultados:**

Participaram 330 estudantes. Identificou-se ausência de correlação entre a atenção plena disposicional e escore geral de regulação emocional e/ou estresse percebido e correlação fraca com a dimensão de regulação emocional supressão das emoções. Estar em tratamento psicológico e uso de substância psicoativas se associou à diminuição da atenção plena disposicional. Já idade, suficiência de horas de sono e supressão emocional se associaram ao aumento desta variável.

**Conclusão:**

Houve relação entre a atenção plena disposicional apenas com a supressão das emoções, além de ligação desta variável com percepção de horas suficientes de sono, idade, uso de álcool ou substâncias psicoativas, estar em acompanhamento psicológico/psiquiátrico e supressão emocional.

## RESUMEN

**Objetivo:**

Investigar la correlación entre mindfulness disposicional, regulación emocional y estrés percibido y verificar factores asociados al mindfulness disposicional en estudiantes de enfermería.

**Método:**

Estudio transversal, correlacional con estudiantes de universidades públicas. Los instrumentos fueron utilizados: Mindful Attention Awareness Scale, Emotional Regulation Questionnaire y Perceived Stress Scale. Los análisis se realizaron mediante pruebas t, correlación de Pearson y regresión lineal múltiple.

**Resultados:**

Participaron 330 estudiantes. No hubo correlación entre mindfulness disposicional y puntuación general de regulación emocional y/o estrés percibido y una débil correlación con la dimensión de regulación emocional supresión de emociones. Estar en tratamiento psicológico y el consumo de sustancias psicoactivas se asociaron con una disminución del mindfulness disposicional. La edad, la suficiencia de horas de sueño y la supresión emocional se asociaron con un aumento de esta variable.

**Conclusión:**

Hubo relación entre el mindfulness disposicional solo con la supresión de emociones, además de la conexión de esta variable con la percepción de suficientes horas de sueño, edad, consumo de alcohol o sustancias psicoactivas, estar en seguimiento psicológico/psiquiátrico y supresión emocional.

## INTRODUCTION

University nursing education tends to be characterized as a potentially stressful experience for some students, because they need to adapt to higher education and deal with stressors common to this period, such as moving away from family and friends circles, taking on new responsibilities and building new interpersonal relationships^(1)^.

Moreover, nursing students must face a range of extra stressors, peculiar to this formative journey, such as dealing with pain, illness and death, events that generate intense emotional stimulus and can manifest in the form of stress. Excessive academic activities and anxiety resulting from simulated or real clinical experiences are also consistent with the increase in the experience of stress, making nursing training an emotionally complex experience^(1)^.

If these students have difficulties to properly deal with emotions, when faced with the myriad of challenges of the training process, they may suffer psychological, cognitive and academic and clinical performance impairment, which can increase the probability of errors, producing poor results in nursing care provided in clinical learning environments, generating new emotional demands on future nurses^(2,3)^. In this sense, it is pertinent for nursing students to use emotional regulation strategies as a way to face the stressors related to the education process and thus learn to deal with the emotions aroused.

Emotion regulation is the ability to tolerate positive or negative emotional reactions, understanding them without excess or downplaying them, as well as controlling or releasing them appropriately^(4,5)^. Emotional regulation is also understood as a set of cognitive and behavioral strategies used as stress coping tools for the regulatory action of emotions^(6)^ and has been widely explored in research with educational applications^(7)^.

Allied to the emotional regulation process, mindfulness, whose name full attention has been most frequently used in Brazilian scientific literature, was scientifically introduced in the West at the end of the 20^th^ century by Jon Kabat-Zinn, who understands it as a psychological process that offers more quality of attention to the experience of the present moment, in addition to involving the consciousness that emerges from this action^(8)^.

As an attribute of consciousness and attention, mindfulness can also be understood as a trait or characteristic inherent to the human being, known as dispositional mindfulness, i.e., people have specific attention levels from birth, even without special training, such as meditation^(9)^. However, this disposition to mindfulness in daily life can be perfected by anyone, even in times of pressure and distraction^(9)^.

In the context of educational research, mindfulness has also been widely explored, mainly because it presents itself as a highly effective cognitive tool to increase students’ ability to regulate emotions and mitigate negative stress-related responses, producing positive results in academic performance^(2,3)^ and boosting psychological well-being outcomes^(10)^.

Thus, considering the complex, potentially stressful and unpredictable nature of nursing education, in which students deal with several demands and emotions simultaneously, and that little is known about factors related to mindfulness and their implications for emotional regulation strategies among nursing students, it was considered essential to assess these relationships in the context of nursing training, a field still relatively little explored in the literature^(11,12)^.

Therefore, this research aimed at investigating the correlation between dispositional mindfulness, emotional regulation and perceived stress and verifying factors associated with dispositional mindfulness among nursing students.

## METHOD

### Design of Study

This is an observational, correlational and cross-sectional study, whose design was guided by the STROBE (STrengthening the Reporting of OBservational studies in Epidemiology)^(13)^.

### Local and Period

The study was conducted at a state public university in midwestern Brazilian between August and September 2020. It is noteworthy that, in this period of time, the COVID-19 pandemic, caused by the SARS-CoV-2 coronavirus, has completed six months since it was declared by the World Health Organization (WHO), through data collection and with impacts on university teaching processes marked mainly by recommendations for social distance, suspension of face-to-face academic activities and teaching practices operationalized in a remote format.

### Population, Sample and Sample Size

The target population consisted exclusively of nursing students enrolled in the referred course on any of the university’s three *campi*. At the higher education institution in question, nursing courses have a full-time load, duration of five years, receiving 80 students annually. The entire student population (N = 981) was surveyed to participate in the study, obtaining compliance of 335 students (34.15% of the population), of which five were excluded from the final sample due to the eligibility criteria. Calculation of sample power for multiple linear regression analysis, operationalized through G Power, version 3.1.9.7, showed that the sample size recruited (n = 330) was sufficient, considering the mean effect size (0.15), level of significance of 95% and sampling power of 80%.

### Eligibility Criteria

Inclusion criteria were considered to be duly enrolled in one of the institution’s nursing courses and to be over 18 years of age at the time of data collection. Any students who failed to respond to the dispositional mindfulness questionnaire, the main outcome of this study, were excluded from the study.

### Data Collection

Data were collected virtually, using an electronic form built using Google^®^ Forms, whose link was sent to participants via e-mail and/or messaging application. Contact with students was made available or intermediated through the coordination of nursing courses of the three *campi* of the university under analysis. Thirty days after the start of data collection, the invitation was reiterated, in the form of a reminder, for those students who had not yet answered and were interested in participating. The Informed Consent Form was made available online, and the respondent should select the dialog box corresponding to the “yes” option, indicating their awareness of the term and their acceptance to participate in the study. At this stage, participants were instructed to indicate an e-mail address, used as a response marker.

### Study Variables and Instruments

The following economic variables were assessed:

- Sociodemographic, academic, health and behavioral characterization: own instrument, prepared by the main author, for application in the context of this study, containing questions about gender, age, marital status, employment status, period of the nursing course, use of psychotropic drugs, consumption of alcoholic beverages or psychoactive substances, if they smoke tobacco, if they undergo psychological or psychiatric follow-up, if they have health comorbidities, if they have hobbies, if they participate in therapeutic or spiritual groups, adequate sleep and physical activity.
- Dispositional mindfulness: measured through the Mindfulness Awareness Scale instrument in its translated version and validated for use in Brazil, with good psychometric performance^(14)^. This is a self-reported, one-dimensional self-report scale, consisting of fifteen questions, with a Likert-type response pattern^(14)^.
- Emotional regulation: assessed using the Emotional Regulation Questionnaire, in its version translated and validated into Brazilian Portuguese, with good psychometric adjustment^(15)^. This is a self-administered, multidimensional instrument, composed of ten questions with a Likert-type response pattern. Six questions of the instrument assess strategies for cognitive reappraisal and four questions assess emotional suppression^(15)^.
- Perceived stress: assessed through the Perceived Stress Scale, in its version translated and validated into Brazilian Portuguese, with good psychometric performance in this process^(16)^. It is a self-administered, one-dimensional instrument that assesses self-perception of stress in the last 30 days. It consists of fourteen items with answers arranged on a Likert-type scale, also analyzed continuously^(16)^.

### Data Analysis and Treatment

Comparative analyzes between mean scores of dispositional mindfulness and dichotomous sociodemographic, academic, health and behavioral variables were performed using t-tests for independent samples, adopting a 95% confidence level. For the only polytomous academic variable (semester studies), one-way analysis of variance (one-way ANOVA) was used to identify differences between groups^(17)^. For the correlational analysis between dispositional mindfulness and scalar variables, Pearson’s product-moment correlation coefficient was used. For these analyses above, 95% confidence intervals (95% CI) were obtained through bootstrapping procedures (1000 resampling), which provide 95% bias-corrected and accelerated bootstrap confidence intervals (95% BCa CI). This technique was used in order to correct deviations from the normality of the sample distribution and differences between the sizes of the groups^(18)^.

Multiple analysis of associated factors was performed using the multiple linear regression technique. For the construction of the final model adopted, all explanatory variables that presented p-value <0.20 in the bivariate analysis were considered. These were introduced individually, one by one, by the ENTER method, being retained in the final model those that presented values of p ≤ 0.05. It is also noteworthy that before adopting the final model, the assumptions for multiple linear regression were checked and met: residue distribution normality; absence of multicollinearity verified through the Variance Inflation Factor (VIF < 10); and non-occurrence of residual autocorrelation using the Durbin-Watson Test.

Analyzes were processed using Statistical Package for the Social Sciences (SPSS), version 23.0.

### Ethical Aspects

The research was approved by the Research Ethics Committee, under Opinion 3,938,455 of March 27, 2020. The Free and Informed Consent Form accompanied the electronic questionnaire, which could only be accessed by those who agreed to participate in the study, following the precepts and ethical guidelines in force in Brazil.

## RESULTS

Data from a composite sample were analyzed by 330 undergraduate nursing students from three different university *campi* of the same public institution of higher education. These students presented moderate scores of dispositional mindfulness (X = 55.89), emotional regulation (X = 48.72) and perceived stress (X = 31.82) (Table 1).

**Table 1.** Mean scores and standard deviation of dispositional mindfulness, emotional regulation, cognitive reappraisal, emotional suppression and perceived stress of nursing students at a public university in midwestern Brazil (n = 330) – Cáceres, MT, Brazil, 2020.

Variable	Scale amplitude	Mean score (standard deviation)	95% BCa CI
Dispositional mindfulness	15 – 90	55.89 (±14.31)	54.41 – 57.33
Emotional regulation (general score)	10 – 70	48.72 (±10.82)	47.63 – 49.82
Cognitive reappraisal	06 – 42	28.80 (±6.84)	28.00 – 29.60
Emotional suppression	04 – 28	19.92 (±5.16)	19.29 – 20.49
Perceived stress	00 – 56	31.83 (±3.79)	31.43 – 32.23

**95% BCa CI:** 95% bias-corrected and accelerated bootstrap confidence intervals.

In the comparative analysis between sociodemographic/academic characteristics and dispositional mindfulness levels, it was found that nursing students enrolled in the headquarters‘ course had higher mean dispositional mindfulness scores when compared to those enrolled in other *campi* (p = 0.024), which was the only statistically significant difference found between this group of variables (Table 2).

**Table 2.** Comparative analysis between sociodemographic/academic characteristics and dispositional mindfulness levels among nursing students at a public university in midwestern Brazil (n = 330) – Cáceres, MT, Brazil, 2020.

Sociodemographic and academic characteristics	Mean score (standard deviation)	Z	P-value
**Sex**			
Female (n = 293)	56.03 (±14.30)	0.167	0.645
Male (n = 37)	54.84 (±14.55)		
**Median age**			
≤ 22 years (n = 194)	54.75 (±13.74)	1.992	0.090
> 23 years (n = 136)	57.52 (±14.99)		
**Marital status**			
With partner (n = 64)	59.11 (±14.99)	0.032	0.800
Without partner (n = 266)	55.12 (±14.06)		
**Employment status**			
Only studying (n = 187)	56.09 (±13.92)	0.424	0.065
Studying and working (n = 143)	55.64 (±14.85)		
**University campus enrolled**			
Headquarters	58.01 (±13.68)	0.242	0.024
Outside headquarters	54.54 (±14.57)		
Course period*			
Initial semesters (1^st^ to 4^th^ semester) (n = 143)	55.77 (±14.30)	2.913	0.056
Intermediary semesters (5^th^ to 8^th^ semester) (n = 132)	54.37 (±14.51)		
Final semesters (9^th^ or 10^th^ semester) (n = 55)	59.87 (±14.12)		

*Comparative analysis performed through one-way ANOVA.

Regarding the health and behavioral characteristics assessed in this study, lower dispositional mindfulness levels were found among students who reported frequent use of alcoholic beverages or other psychoactive substances (p = 0.019), reported having psychiatric and/or psychological follow-up (p = 0.001), reported using medication for therapeutic purposes (p = 0.002) and with a subjective perception of insufficient sleep hours (p = 0.001) (Table 3).

**Table 3.** Comparative analysis between health/behavioral characteristics and dispositional mindfulness levels among nursing students at a public university in midwestern Brazil (n = 330) – Cáceres, MT, Brazil, 2020.

Health and behavioral characteristics	Mean score (standard deviation)	Z	P-value
**Tobacco use**			
No (n = 319)	55.93 (±14.35)	0.246	0.810
Yes (n = 11)	54.91 (±13.77)		
**Frequent consumption of alcoholic beverages or other psychoactive substances**			
No (n = 255)	56.94 (±14.38)	0.778	0.019
Yes (n = 75)	52.35 (±13.57)		
**Psychiatric and/or psychological follow-up**			
No (n = 290)	57.31 (±14.00)	1.92	0.001
Yes (n = 40)	45.60 (±12.30)		
**Use of psychotropics for therapeutic purposes**			
No (n = 393)	56.76 (±14.04)	0.006	0.002
Yes (n = 37)	49.00 (±14.70)		
**Health comorbidities (self-report of two or more simultaneous problems)**			
No (n = 304)	56.30 (±14.26)	0.036	0.081
Yes (n = 26)	51.12 (±14.25)		
**Subjective perception of sufficient sleep hours**			
Sleeping sufficient hours (n = 154)	59.24 (±13.30)	1.106	0.001
Not sleeping sufficient hours (n = 176)	52.94 (±14.54)		
**Physical activity**			
No (n = 149)	55.18 (±14.03)	0.605	0.415
Yes (n = 181)	56.48 (±14.54)		
**Participation in groups (therapeutic, musical, artistic and/or spiritual)**			
No (n = 252)	56.08 (±14.58)	0.070	0.851
Yes (n = 78)	55.31 (±13.47)		
**Hobby/hobbies**			
No (n = 78)	56.17 (±14.15)	0.409	0.666
Yes (n = 252)	55.81 (±14.38)		

Table 4 presents a correlational analysis between dispositional mindfulness, emotional regulation, cognitive reappraisal, emotional suppression and perceived stress. A weak and positive correlation was found between dispositional mindfulness and the emotional suppression score (r = 0.125), indicating that the higher the dispositional mindfulness levels, the higher the indicators of this emotional regulation dimension. Moreover, weak and positive correlations were also found between perceived stress and all the indicators of emotional regulation adopted in this study.

**Table 4.** Correlational analysis between dispositional mindfulness levels, emotional regulation and perceived stress variables among nursing students at a public university in midwestern Brazil (n = 330) – Cáceres, MT, Brazil, 2020.

	Dispositional mindfulness	Emotional regulation	Cognitive reappraisal	Emotional suppression	Perceived stress
Dispositional mindfulness	–				
Emotional regulation	0.092 (–0.02; 0.19)	–			
Cognitive reappraisal	0.052 (–0.05; 0.15)	0.927* (0.91; 0.94)	–		
Emotional suppression	0.125* (0.02; 0.22)	0.868* (0.84; 0.89)	0.619* (0.54; 0.68)	–	
Perceived stress	–0.076 (–0.17; 0.02)	0.160* (0.05; 0.27)	0.137* (0.03; 0.24)	0.154* (0.04; 0.26)	–

In parentheses, 95% bias-corrected and accelerated bootstrap confidence intervals; *significant result obtained via bootstrapping were presented.

Table 5 presents the variables that remained associated with dispositional mindfulness after applying the multiple linear regression analysis method, highlighting that the final model adopted was statistically significant [F(6,335) = 11.697; p < 0.001, R^2^= 0.178], indicating that the set of retained variables was able to explain 17.8% of the outcome variance.

**Table 5.** Factors associated with dispositional mindfulness from a multiple linear regression model in a sample of nursing students at a public university in midwestern Brazil (n = 330) – Cáceres, MT, Brazil, 2020.

Variables	Multiple linear regression analysis						
	Non-standardized coefficients		Standardized coefficient	95%CI	t	P-value	VIF
	B	Standard error	β				
Intercept	48.161	6.7890		34.805; 61.517		< 0.001	
Psychological follow-up (yes)	–11.011	2.236	–0.252	–15.409; –6.612	–4.925	< 0.001	1.026
Sufficient sleep hours (yes)	5.776	1.452	0.202	8.633; 2.919	2.977	< 0.001	1.011
Age	0.488	0.123	0.186	0.206; 0.690	3.647	< 0.001	1.023
Use of alcohol or psychoactive substances (yes)	–3.891	1.731	–0.114	–7.296; –0.487	–2.248	0.025	1.013
Emotional suppression	0.308	0.143	0.111	0.027; 0.588	2.158	0.032	1.040
Perceived stress	–0.298	0.195	0.079	–0.682; 0.087	1.523	0.129	1.055

Notes: method of insertion: ENTER; R^2^: 0.178; F (6.335): 11.697 (p < 0.001); Durbin-Watson (2.161)

The variable psychological or psychiatric follow-up/ treatment was the one that presented the greatest explanatory power within this model (β= – 0.252, t = – 4.925, p < 0.001), demonstrating that students under this type of professional follow-up presented losses of 11.011 points on the dispositional mindfulness scale. The subjective perception of sufficient sleep hours (β = 0.202, t = + 2.977, p < 0.001) was also identified, which was associated with increased dispositional mindfulness levels, and the use of psychoactive substances (β = – 0.114, t = – 2.248, p = 0.025), which, in turn, was associated with decreased dispositional mindfulness.

In addition to these variables, age (β = 0.186, t = 3.647, p < 0.001) and the emotional suppression subscale (β = 0.111, t = 2.158, p = 0.032) were also factors associated with dispositional mindfulness among nursing students, noting that both were positively associated with this measure, i.e., as age and emotional suppression scores increased, the dispositional mindfulness indicator used increased concomitantly. It is also noteworthy that perceived stress (β = 0.079, t = 1.523, p = 0, 129) was maintained in the final model as an adjustment variable, without which the statistical significance of emotional suppression was lost and the explanatory capacity of the model was reduced.

## DISCUSSION

This study assessed dispositional mindfulness levels and its relationships with indicators of emotional regulation, perceived stress, sociodemographic, academic, health-related and behavioral characteristics in a sample of undergraduate nursing students. Regarding the dispositional mindfulness levels, this sample presented a mean score consistent with what has been reported in studies with similar outcomes and population. However, it is important to highlight that recent studies that assessed dispositional mindfulness among nursing students through the Mindful Attention Awareness Scale showed wide variability in the mean scores reported in their baseline studies (pre- interventions), ranging from 45.80^(19)^ to 60.51^(20)^ points. This is possibly due to the academic and sociocultural distinctions of each assessed university context.

Moreover, the results of this study did not confirm the hypothesis of a positive correlation between dispositional mindfulness and emotional regulation levels among nursing students, which was expected, given that, as pointed out in the literature^(11,12)^, mindfulness represents an important mechanism of emotional regulation, acting in transforming negative emotions^(21)^ and allowing individuals to more accurately decipher their own emotions or those of others, equipping them with better capacities to manage them, mainly through non-judgmental and self-regulating aspects of mindfulness itself^(12)^. The absence of significant correlation between these two variables was not exclusive to this study, and was in agreement with the findings of a study conducted with Italian nurses^(21)^.

Regarding the two sub-dimensions of emotional regulation assessed, there was no significant correlation between cognitive reappraisal and dispositional mindfulness, even though this variable is considered the operating mechanism of mindfulness interventions^(22)^. The emotional suppression dimension correlated, albeit weakly, positively with dispositional mindfulness, demonstrating that an increase in any of the variables resulted in an increase in the other’s mean scores. This finding was divergent from previous evidence, which found no significant correlation between emotional suppression and dispositional mindfulness^(11)^, and different from a study that pointed out these indicators correlating inversely^(22)^.

The weak correlations or the absence of correlations between dispositional mindfulness and emotional regulation variables can be explained by the instrument used to measure the outcome. A meta-analysis study showed that studies that correlated mindfulness and emotional regulation using the Mindful Attention Awareness Scale were those that showed the smallest magnitudes of effect in these relationships, when compared to studies that assessed mindfulness using other instruments. These authors concluded that this could be due to the fact that the Mindful Attention Awareness Scale, in their assessment, does not emphasize emotional domains, as occurs, for instance, with other scales, such as the Five Facet Mindfulness Questionnaire (FFMQ) and the Freiburg Mindfulness Inventory (FMI)^(12)^.

In support of these results, a review article summarized the existing relationships between mindfulness and emotional regulation, identifying that, currently, there is no consensus in the scientific literature about the concept and the measures used to assess this measure. Furthermore, the study reinforces the existence of overlapping factors that both constitute dispositional mindfulness and emotional regulation, which could hinder the clear identification of relationships between them^(10)^.

Regarding the associated factors identified in this study, the fact that students who reported undergoing psychological or psychiatric follow-up had decreased dispositional mindfulness scores may not be related to the act of properly seeking specialized help for mental health, but because it is mediated by other variables not measured in this study, such as type, regularity and duration of psychological or psychiatric treatment used, or even type, dose and duration of use of psychotropic drugs used, which may contribute to a decrease in dispositional mindfulness levels^(23)^.

The assessment of the subjective perception of sleep sufficiency reported by students showed that having sufficient sleep to meet individual sleep needs seems to increase nursing students’ capacity to remain more attentive to the events of the present moment and act with more awareness. The perception of insufficient hours is a relatively prevalent experience among undergraduate students^(24)^, and its genesis may be related mainly to the high stress levels experienced by these individuals, resulting from the pressures, demands and academic expectations that trigger disturbances in sleep pattern^(24)^.

The sample of this study consisted of mostly young nursing students, with age having a significant relationship with dispositional mindfulness indicators presented by them, showing better scores for this variable among older students and improvement over the years. Previous evidence suggests that, over the years, people tend to develop greater emotional control/management skills and improve their ability to act with more awareness and be more attentive to the events of the present moment, once they are no longer interrupted by the intensity of their emotions, being natural processes of human development^(25)^.

As for self-reported use of alcohol and/or psychoactive substances, these variables were associated with decreased dispositional mindfulness levels, corroborating evidence from other studies, which have shown that lower dispositional mindfulness levels are associated with a higher probability of becoming users of psychoactive substances^(26)^ and greater severity of dependence on these substances^(27)^. Although this study does not differentiate the frequency of use of these substances, distinguishing sporadic from chronic users, it is recognized that the use of psychoactive substances tends to impair conscientiousness and clarity of emotions, provoke automatic or unreflective behaviors and increase the experience of negative emotions and use of dysfunctional emotional regulation strategies^(27)^.

The emotional suppression variable also proved to be a factor associated with dispositional mindfulness in this sample of nursing students, although in a different way than initially conjectured. The use of emotional suppression, in an attempt to avoid or escape the experience and awareness of one’s own emotions, has been seen by researchers as a dysfunctional and maladaptive emotional regulation strategy^(6)^, often being inversely associated with indicators of mindfulness^(22)^. From a neurobiological perspective, It has been suggested that suppressing emotions promotes exaggerated activity of the sympathetic nervous system that can affect immune responses and physical health, acting physiologically contrary to mindfulness, increasing parasympathetic nervous system activity and, in essence, alleviating systemic agitation^(6)^.

However, there are already studies that demonstrate that emotional suppression may not always manifest itself as a strategy of dysfunctional emotional regulation, and may be effective for certain individuals in certain contexts, requiring a careful assessment of each situation^(28)^, however further studies still seem necessary for a better understanding of these processes^(10)^.

Finally, it is important to highlight the relationships established between perceived stress and dispositional mindfulness and the variables related to emotional regulation. Perceived stress was not associated with dispositional mindfulness in this sample of nursing students. However, was retained in the final model for adjustment purposes, improving the explanatory capacity of the model and ensuring the statistical significance of the variable emotional suppression, which may denote, even though stress did not directly impact dispositional mindfulness levels, that it may have acted indirectly, increasing emotional suppression levels, which, in turn, impacted dispositional mindfulness. Although not consensual^(29)^, the literature has pointed out an association between high perceived stress levels and the greater use of emotional regulation strategies considered dysfunctional, such as emotional suppression and avoidance^(30)^.

## LIMITATIONS

First, the cross-sectional design, although capable of providing important information for the advancement of scientific knowledge, has limitations because, in this design, all variables are measured simultaneously, with no structural distinction between predictor variables and outcome variables, which limits conclusions about causality or temporal precedence. Furthermore, the convenience sample and the fact that the study was carried out on different *campi* of a single higher education institution may not reflect the representative characteristics of the entire population. Future studies may cover a broader sample base and include nursing undergraduates from different institutions, regions or countries.

## CONCLUSION

This sample of students presented moderate mean scores of dispositional mindfulness, emotional regulation and perceived stress. There was no significant correlation between dispositional mindfulness, the general emotional regulation score and perceived stress, with this first variable being weakly correlated with the emotional regulation emotional suppression dimension. In addition to this, being in psychological treatment and use of psychoactive substances were associated with a decrease in dispositional mindfulness levels, whereas age, sufficient sleep hours and emotional suppression were associated with an increase in it.

Thus, the results indicate a relationship between dispositional mindfulness only with the emotional regulation emotional suppression dimension, in addition to the connection of dispositional mindfulness with the variables perception of sufficient sleep hours, age, use of alcohol or psychoactive substances, being in psychological/psychiatric follow-up and emotional suppression.
